# Ancient DNA: the past for the future

**DOI:** 10.1186/s12864-023-09396-0

**Published:** 2023-06-08

**Authors:** Ningbo Chen, Artem Nedoluzhko

**Affiliations:** 1 Key Laboratory of Animal Genetics, Breeding and Reproduction of Shaanxi Province, College of Animal Science and Technology, Northwest A&F University, Yangling, 712100 China; 2 Paleogenomics Laboratory, European University at St. Petersburg, 6/1A Gagarinskaya Street, St. Petersburg, 191187 Russia

## Abstract

The last decade has seen advancements in sequencing technologies and laboratory preparation protocols for ancient DNA (aDNA) that have rapidly been applied in multiple research areas thus enabling large-scale scientific research. Future research could also refine our understanding of the evolution of humans, non-human animals, plants, invertebrate specimens, and microorganisms.

## Main text

aDNA is genetic material that can be extracted from the vast majority of ancient biological remains dating to the last tens of thousands of years. Certain types of ancient biological material (bones, hair bulbs, skin fragments, fish scales, teeth, and sediments, etc.) are able to preserve DNA molecules for a long time. However, aDNA, like any chemical substance, interacts with the environment, degrades into short DNA fragments (approximately 50–100 base pairs (bp)), undergoes postmortem mutations (e.g., cytosine deamination to uracil), and mixes with the environmental DNA (DNA contamination). Such limitations complicate aDNA studies and result in the necessity of additional methodical approaches during the analyses [1]. Nevertheless, aDNA research began in 1984 with the extraction and sequencing of short mitochondrial fragments from the extinct quagga (*Equus quagga quagga*) [2]. This was immediately followed by a paper in which Svante Pääbo published the first sequence of ancient human DNA, 340 bp in length, isolated from an Egyptian mummy [3]; in 2022, he was awarded the Nobel Prize for his incredible discoveries concerning the genomes of extinct hominins and human evolution [4].

Four decades later, with technical advances in aDNA extraction, sequencing, and bioinformatics methods, aDNA data have also considerably enhanced our understanding in anthropology, evolutionary biology and archaeological sciences. A milestone that paved the way to such accomplishments was the introduction of next-generation sequencing techniques in the aDNA fields. Genome-scale data have now been gathered from thousands of paleobiological species, and the number of ancient biological tissues amenable to genome sequencing is growing steadily [1]. Every week, aDNA studies expand our knowledge about the past.

The application of aDNA analysis is broad in scientific scope and relevant to key archaeological, ecological and evolutionary questions. Multiple areas have received heightened scholarly attention, including human population history, plant and animal domestication, and the origins and evolution of pathogens and microbiomes. Interdisciplinary collaborations have also been established, and more scholars are being trained to reconstruct archaeogenetic prehistory in more integrative and sophisticated ways.

The abundance of paleogenomic data from ancient humans, some obtained with the application of DNA capture techniques, has allowed detailed insights into the rich history of admixture between early modern humans, Neanderthals, and Denisovans and has helped disentangle evolutionary origins, migrations, interactions, and disappearances of past human populations that were previously inaccessible. More than 6,000 ancient genomes, most of them from Northern Eurasia, have been reconstructed to date, but we have only scratched the surface of human history [5]. Efforts should be focused on obtaining ancient genomes in Africa, Asia, the Americas, and Oceania [5].

The domestication of animals and plants led to a major shift in human subsistence patterns. Studies on ancient plant and animal DNA have lagged behind those on human subsistence patterns, from a hunter-gatherer to a sedentary agricultural lifestyle, which ultimately resulted in the development of complex societies [6]. Over the past decade, ancient genomics in various domesticated organisms, such as dogs, pigs, goats, cattle, carp species, Nile tilapia, bread wheat, barley and maize, has begun to dramatically shift our understanding of the process of domestication [6]. Although a great deal of progress has been made in understanding animals and plants domestication, several key questions remain, with several fundamental questions regarding wild progenitor species, time of domestication and geographic regions of domestication yet to be answered. It is estimated that approximately 2,500 species of plants have been subject to domestication since the last glacial period, over 12,000 years ago [7]. However, the domestication history of most crop species and some animals, such as yak and water buffalo, remains unclear.

Our experience in molecular biology, paleogenomics, and bioinformatics allows to conduct research focused on the conservation of endangered species and even de-extinction projects, which have recently attracted considerable attention from the near-scientific and scientific communities. In 2021, the biotech company Colossal (https://colossal.com/) announced its plans to use genetic engineering to resurrect the woolly mammoth (*Mammuthus primigenius*) and return it to the Arctic tundra, its original natural habitat. Mammoths are not their only goal; they are also planning the “resurrection” of the passenger pigeon (*Ectopistes migratorius*), the Tasmanian tiger (*Thylacinus cynocephalus*) and the dodo bird (*Raphus cucullatus*). Moreover, other scientific groups are going to restore the Christmas Island rat (*Rattus macleari*), which disappeared just over 100 years ago [8] and Steller’s sea cow (Hydrodamalis gigas) which was extinct approximately 250 years ago (Arctic Sirenians Project). Nevertheless, the de-extinction projects are not as simple as they seem at first glance. To date, more than twenty nuclear genomes of extinct animal species, such as woolly mammoth, cave lion (*Panthera leo spelaea*), moa (*Dinornis novaezealandiae*) and other, have been sequenced; however, none of these species has been restored yet. Furthermore, de-extinction projects are on the edge of ethical principles since nobody knows how reviving species will influence modern ecosystems [9].

Despite unprecedented achievements in our ability to mine high-quality ancient genomic information from multiple samples with varied preservation statuses, current paleogenomic techniques are still far from sufficient to offer a thorough understanding of the genetic history of humans, plants, non-human animals, and micro-organisms. However, there is still much to discover and the continued technological and computational developments in the aDNA field have transformed our understanding of the past and will surely continue to uncover future mysteries.

**Fig. 1:**
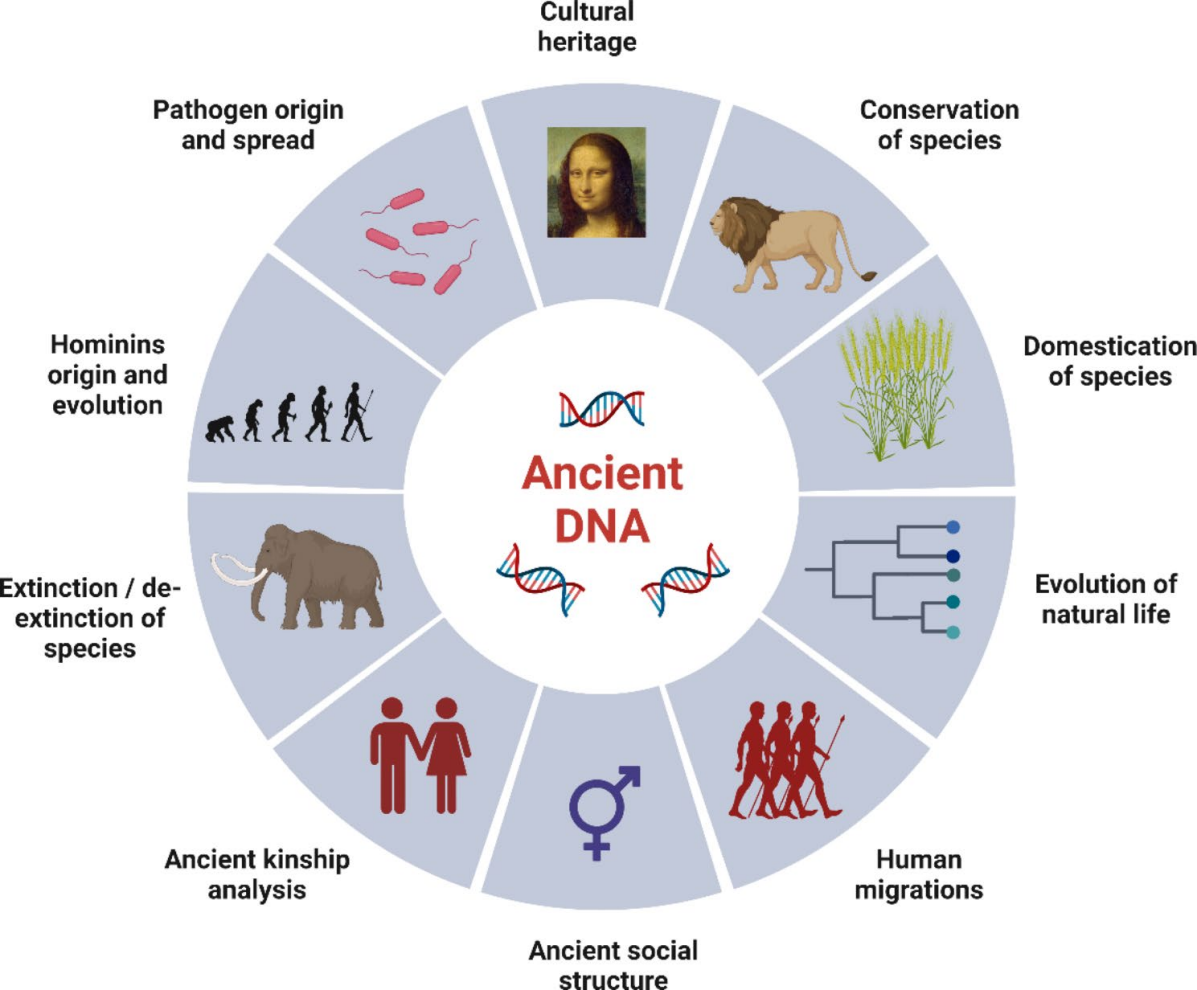
Possible areas of ancient DNA method implementation for anthropological and paleobiological material

With this Ancient DNA Collection in BMC Genomics and BMC Genomic Data we hope to see work in the broad field of aDNA (Fig. 1) and focused on the implementation of paleogenomics methods as follows:

1. DNA extraction and experimental workflow;
2. Archaic hominin species origin and evolution;
3. Evolution of plants and animals;
4. Conservation studies;
5. Pathogen origin and evolution;
6. Cultural heritage studies;
7. Domestication of species;
8. Human migrations, the history of ancient societies, and kinship analysis;
9. Extinction and de-extinction of species.

Bioinformatic tools enabling re-evaluation of previously published data and novel computational approaches and pipelines for ancient DNA analysis are also welcome and BMC Genomic Data encourages the submission of ancient DNA Data Notes.

## Data Availability

Not applicable.
